# Prevention and Treatment of Side Effects of Immunotherapy for Bladder Cancer

**DOI:** 10.3389/fonc.2022.879391

**Published:** 2022-05-20

**Authors:** Kecheng Lou, Shangzhi Feng, Guoxi Zhang, Junrong Zou, Xiaofeng Zou

**Affiliations:** ^1^ The First Clinical College, Gannan Medical University, Ganzhou, China; ^2^ Department of Urology, The First Affiliated Hospital of Gannan Medical University, Ganzhou, China; ^3^ Institute of Urology, The First Affiliated Hospital of Gannan Medical University, Ganzhou, China; ^4^ Jiangxi Engineering Technology Research Center of Calculi Prevention, Gannan Medical University, Ganzhou, Jiangxi, China

**Keywords:** bladder cancer, immunotherapy, immune checkpoints, immune-related adverse events, targeted immunotherapy

## Abstract

Bladder cancer (BC) is one of the most important tumors of the genitourinary system, associated with high morbidity and mortality rates. Over the years, various antitumor treatments have been developed, and immunotherapy is one of the most effective methods. Immunotherapy aims to activate the body’s immune system to kill cancer cells. It has been established that immunotherapy drugs can be classified into “non-targeted” and “targeted” drugs depending on their site of action. Immunotherapy is reportedly effective for BC. Even though it can attack cancer cells, it can also cause the immune system to attack healthy cells, which can occur at any time during treatment and sometimes even after immunotherapy is stopped. Importantly, different types of immunotherapies can cause different side effects. Side effects may manifest themselves as signs or as symptoms. The prevention and treatment of side effects caused by immunotherapy is an important part of cancer patient management.

## Introduction

BC is among the top ten most common cancer types in the world, according to an observatory in 2018, with approximately 55000 new cases and 200000 deaths annually (1). It ranks tenth in worldwide absolute incidence: sixth in men and seventeenth in women (2). The worldwide Age Standardized Incidence Rate per year (ASR) is 9.6 per 100000 for males and 2.4 per 100000 for females (3).

Smoking is the most significant risk factor of BC, associated with 50-65% of male cases and 20-30% of female cases. The incidence of BC is reportedly directly associated with the duration of smoking, and the number of cigarettes smoked per day (4). Occupational factors are the second most important risk factor for BC (5).

Uroepithelial carcinoma originating from the bladder is the most common histologic type of cancer. Over 70% of cases are diagnosed at the non-muscle invasive stage and managed by minimally invasive local treatment. Unfortunately, this disease has a high recurrence rate and may require further treatment with more than one modality. In contrast, the muscle-invasive and metastatic stage of the disease requires multimodal treatment strategies, including surgical treatment and chemotherapy in addition to neoadjuvant, adjuvant or palliative care (6).

Cancer therapies that alter the immune status have gained prominence in oncology in recent years (7). Immunotherapy is often used to complement traditional cancer treatments such as surgery, chemotherapy, and radiation therapy. During clinical practice, it is used as a first-line treatment for some cancers (8) and involves the patient’s immune system to modify or increase the defense mechanisms against the developing cancer cells (8). The first clinical application of immunotherapy was documented in the 1890s when William Coley first used a bacterial agent called Coley’s toxin. Clinical trials showed minimal results. Importantly, this toxin provided the first compelling evidence of the potential to produce an antitumor response using the patient’s immune system (8). Immunotherapy became part of standard cancer treatment in the mid-20th century, although it exhibited significant toxicity. Treatment with cell therapy and the development of bone marrow transplantation was initiated by Fritz Bach et al. in the 1960s, as well as the production, testing and approval of high doses of IL-2 (interleukin 2) for the treatment of metastatic kidney cancer and melanoma in clinical trials in the 1990s (9, 10). Several types of immunotherapies are currently used to treat cancer, including immune checkpoint inhibitors, T-cell transfer therapy, monoclonal antibodies, therapeutic vaccines, and immune system modulators.

Immunotherapy has an anti-cancer effect because it activates the immune response against cancer cells more specifically and strongly, thus killing them. In tumors, mutated or dysregulated proteins are processed into peptides, then loaded onto major histocompatibility complex I (MHCI) molecules to form immune complexes recognized by CD8+ T cells (11). Then cytotoxic T lymphocytes are activated (12), which not only kill cancer cells and inevitably cause some damage to normal cells, but may eventually attack any of the body’s healthy or normal tissues or organs, leading to unpredictable side effects, also known as “immune-related adverse events (irAE)”. Organ specificity, incidence, and severity of irAEs vary according to each agent and its dose, but also differ across tumor types (13). Immune-related adverse events include non-specific symptoms and damage to the skin and mucous membrane system, head and five sense organs, digestive system, cardiovascular system, respiratory system, endocrine system, blood system, neuropsychiatric system, bone and joint system, and immune system (14). Immunotherapy has benefited a significant proportion of BC patients and has even been able to cure cancer in some patients in combination with other drugs. This new treatment modality offers hope to cancer patients but emphasizes that the associated toxic side-effects are currently a challenge for effective clinical treatment (**Table 1**)

**Table 1:** List of side effects, indications and serious complications for immunotherapy for bladder cancer.

Compound	Target	Side effects	Serious Complications	Clinical Indications	Reference
BCG	Non-Target	Digestive, urinary, skeletal joint problems, and general symptoms.	Sepsis and pneumonia.	Carcinoma in situ, high-grade papillary tumors, and invasive plaque-proprious tumors.	(15)
The mTOR Kinase Inhibitors	Non-Target	Digestives, hematologic, dermatomycoses, endocrine problems, and general symptoms.	Cardiac insufficiency, respiratory failure and sepsis.	For adult patients with unresectable, locally advanced, or metastatic disease with progressive neuroendocrine tumors of gastrointestinal or pulmonary origin.	(16)
COX-2 Inhibitors	Non-Target	Digestive, cardiovascular system, urinary problems, and general symptoms.	Peptic ulcer.	Mainly used for the prevention of bladder cancer.	(17, 18)
Nivolumab	PD-1	Digestive, urinary, respiratory, dermatomycoses, endocrine problems, and general symptoms.	Infusion reaction, intestinal obstruction, urinary tract and infection, sepsis.	Locally advanced or metastatic uroepithelial carcinoma.	(19, 20)
Pembrolizumab	PD-1	Digestive, urinary, respiratory, dermatomycoses, endocrine, skeletal joint problems, and general symptoms.	Pneumonia and cardiac insufficiency.	BCG-non-responsive, high-risk, non-muscle-invasive bladder cancer patients (NMIBC) with carcinoma *in situ* (CIS) with or without papillary tumors who are not candidates for or have chosen not to undergo cystectomy.	(21–23)
Durvalimab	PD-L1	Digestive, urinary, skeletal joint problems, and general symptoms.	Peptic ulcer.	Patients with locally advanced or metastatic uroepithelial carcinoma.	(24, 25)
Atezolizumab	PD-L1	Digestive problems, urinary problems, immune problems, and general symptoms.	Pneumonia, drug hepatitis, colitis, intestinal obstruction, endocrine diseases, and pancreatitis.	Patients with locally advanced or metastatic urothelial carcinoma that experience exacerbations during or following platinum-containing chemotherapy, or within 12 months of receiving platinum-containing chemotherapy, either before (neoadjuvant) or after (adjuvant) surgical treatment.	(26–28)
Avelumab	PD-L1	Skeletal joint, endocrine, dermatomycoses, digestive, urinary, respiratory problems, and general symptoms.	Infusion reaction, pneumonia, colitis, drug hepatitis, nephritis, renal insufficiency, and respiratory failure.	Patients with locally advanced or metastatic uroepithelial carcinoma.	(29–31)
Ipilimumab	CTLA-4	Dermatomycoses, neurological, psychiatric and digestive problems.	Peptic ulcer.	–	
Tremelimumab	CTLA-4	–	–	–	
CAR-T	–	Hematologic problems and Immune problems.	–	–	

## Immunotherapy Drugs and Side Effects

### Non-Targeted Immunotherapy Drugs

#### Bacillus Calmette–Guerin

It is widely acknowledged that Everolimus (Afinitor) is an attenuated strain of Mycobacterium Bovis. Although it has been discovered for decades, its exact mechanism of action remains unknown (32). BCG is used as a vaccine and is now used stably in patients with carcinoma *in situ* or moderate or high non-muscle invasive BC (33). It has been shown that BCG can cause a massive release of cytokines and chemokines after attachment to tumor cells by fibronectin and then internalization into tumor cells (34). BCG also promotes tumor antigen presentation to cells of the immune system (35, 36), and induction of long-term adaptive immunity (32, 37). It has been shown that BCG treatment elicits an inflammatory response involving different immune cell subsets, including CD4+ and CD8+ lymphocytes (38, 39), natural killer (NK) cells (40), granulocytes (40, 41) and macrophages (42, 43), among other cell subsets. *In vitro* experiments have shown that integrin cross-linking of BCG leads to cell cycle arrest at the G1/S interface in proliferating cells of human urothelial carcinoma cells, resulting in a direct cytostatic effect on the cancer cell line (44).

BCG is currently the most common and important tool in treating and preventing different forms of superficial BC. In this regard, treatment with BCG after transurethral resection of bladder tumor (TUPRBT) reduces the risk of tumor recurrence or high-grade tumor development, and this is now standard practice in the treatment of non-muscle invasive bladder cancer (NMIBC, including carcinoma in situ, high-grade papillary tumors, and invasive plaque intrinsic tumors) (45). Indeed, BCG treatment is also associated with concomitant side effects. Currently, side effects such as fatigue, fever, mild lower urinary tract symptoms and frank hematuria have been reported in the literature after BCG intravesical infusion therapy for BC (46). Additional side-effects include infections such as granulomatous inflammation of the genitourinary tract (bladder, testes, or prostate), pneumonia, arthritis, and hepatitis. Indeed, tuberculosis may take years to be expressed clinically and often presents as local discomfort, recurrent fever, and night sweats. If the infection worsens, severe systemic manifestations such as high fever, hypotension, organ failure, or septic shock may be observed. Therefore, the BCG vaccine should be used in the prescribed concentration range as much as possible, which will not only increase its effectiveness but also reduce the side effects to some extent (47).

#### The mTOR Kinase Inhibitors

Studies on the use of the mTOR Kinase Inhibitors for BC are ongoing. An increasing body of evidence shows that these drugs act by binding to the tacrolimus binding protein 12 (FKBP-12) protein, forming a complex that inhibits mTOR activity. This phenomenon leads to cell cycle arrest and inhibition of angiogenesis, proliferation, and glucose delivery to cells (48). Angiogenesis is inhibited by downregulated expression of hypoxia-inducible factor 1, which reduces the levels of vascular endothelial growth factor (49). In 2016, the Food and Drug Administration (FDA) approved everolimus for adult patients with unresectable, locally advanced or metastatic disease with progressive neuroendocrine tumors of gastrointestinal or pulmonary origin (50). The most common side effects of this class of drugs include stomatitis, rash, fatigue, hyperglycemia, hyperlipidemia, and myelosuppression; most of these are mild and disappear with drug interruption or dose reduction.

#### COX-2 Inhibitors

Cyclooxygenase inhibitors are compounds that have inhibitory effects on cyclooxygenase. Cyclooxygenase inhibitors include two major groups: nonspecific cyclooxygenase inhibitors, which can inhibit both COX-1 and COX-2, such as aspirin and specific COX-2 inhibitors, such as celecoxib. Interestingly, the Cyclooxygenase-2 (COX-2) inhibitor has been shown to exhibit chemopreventive activity against various cancers, including BC, by inhibiting the proliferation, migration, invasion, and epithelial-to-mesenchymal transition of BC cells. However, its mechanism of action is not fully understood (51). Common adverse reactions mainly involve the digestive, cardiovascular, and urinary systems. Other adverse reactions include systemic reactions, which are generally mild.

### Targeted Immunotherapy Drugs

**Figure 1:**
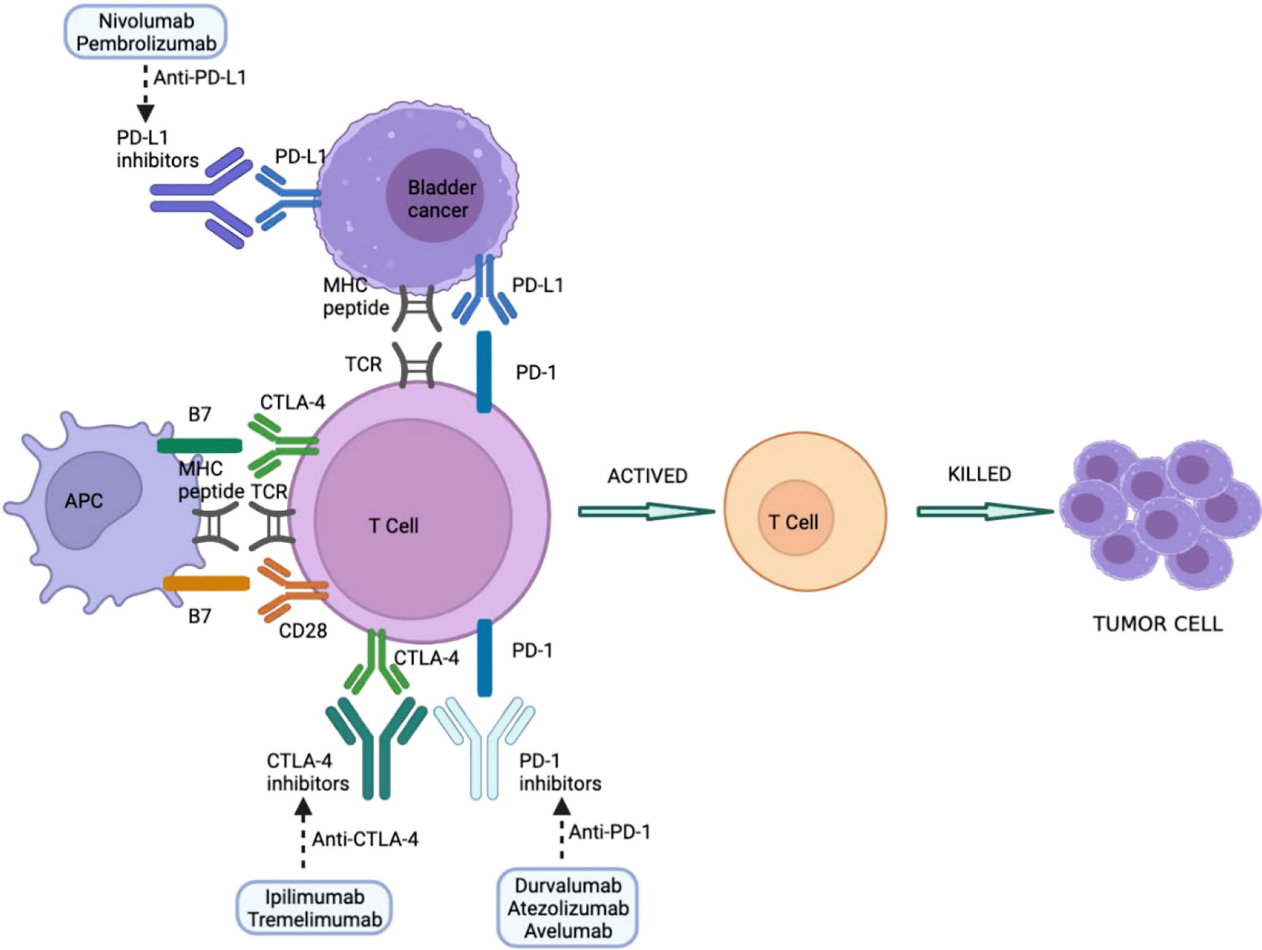
Immune checkpoint inhibitors in BC treatment. PD-1/PD-L1 and CTLA-4 blockers interfere with suppression of checkpoint molecules of the immune system, leading to T-cell activation and tumor cell killing. CTLA-4, cytotoxic T-lymphocyte antigen 4; MHC, major histocompatibility complex; PD-1, programmed cell death-1; PD-L1, programmed cell death-1 ligand; TCR, T cell receptor; APC: Antigen-presenting cell.

Immune checkpoints are molecules involved in maintaining immune homeostasis and therefore contribute to maintaining peripheral tolerance to their own molecules. The main immune checkpoint inhibitors include blockade of programmed cell death protein-1/programmed cell death protein ligand 1 (PD-1/PD-L1) and cytotoxic T cell antigen (CTLA4). The use of monoclonal antibodies that block co-inhibitory immune checkpoint molecules helps to increase T cell-specific immune responses and thus harness the immune system against tumors (52). Responsiveness to checkpoint inhibitors is key to treatment, but this does not necessarily mean that all patients have good outcomes since some can also experience drug side effects. Other immune cells can also play an important role in developing irAEs, including B cells, which can secrete antibodies to conduct toxicity (53, 54), and granulocytes, which secrete inflammatory mediators and cytokines (53, 55). Indeed, it should be borne in mind that the side effects of a drug may not significantly alter its effectiveness; however, the patient’s quality of life may be affected during treatment. Overall, side effects associated with anti-PD-1/PD-L1 are less common and severe than with anti-CTLA-4 antibodies (56). The most typical manifestations involve the skin, gastrointestinal tract, liver, and endocrine system (57). Cutaneous toxicity is the most common irAE, although GI involvement is usually more clinically relevant because of its potential morbidity and management, requiring steroids and hospitalization (58). Other rarely reported irAEs include uveitis, conjunctivitis, neuropathy, myopathy, pancreatitis, pneumonia, hemocytopenia, and nephritis (57). Immune-related adverse events associated with a certain immune checkpoint inhibitor is usually consistent across tumor types (**Figure 1**)

#### PD-1/PD-L1

PD-1 and PD-L1 are important immune checkpoints that negatively modulate the immune system, impairing its response to antigens. PD-1 is expressed on the surface of activated T and B lymphocytes and macrophages, and PD-L1 on antigen-presenting cells (59). The binding of PD-1 and PD-L1 blocks the activation of T lymphocytes, thereby reducing the production of IL-2 (interleukin 2) and interferon-gamma (59). Anti-PD-1 and PD-L1 drugs can block either of these two molecules, preventing both from binding, thereby increasing the production of both cytokines (60).

##### Nivolumab

Nivolumab, a human monoclonal antibody of IgG4 type, was approved by the FDA in 2017 for use in advanced BC (61). The common complications are elevated lipase and amylase, fatigue, skin rash, dyspnea, neutropenia, and lymphopenia (62–64).

##### Pembrolizumab

Pembrolizumab is a humanized IgG4/kappa monoclonal antibody that can be used to treat various types of cancer. Approved by the FDA in 2019 for the treatment of BC, especially for advanced BC cases (21, 65), pembrolizumab may be used as a first-line treatment for uroepithelial cancer (66). Moreover, it can be used to treat patients with DNA repair defects (67), and the reported overall survival with pembrolizumab is significantly higher than with chemotherapy drugs (68). Importantly, Pembrolizumab has a better safety profile than other drugs, although it may cause immune-related adverse effects such as myocarditis and myasthenia gravis.

##### Durvalumab

Durvalumab is an IgG1k monoclonal antibody approved by the FDA to treat BC in 2017. Studies have shown that although Durvalumab has high activity in PD-L1-positive and negative patients, it exhibits relatively higher efficacy in patients with high PD-L1 expression (69).

##### Atezolizumab

Atezolizumab, a humanized IgG1 isotype monoclonal antibody (70), was the first PD-1/PD-L1 checkpoint inhibitor approved by the FDA and is often used in the second-line treatment of patients with advanced BC (71). Most treatment-related adverse events are mild to moderate, including fatigue, nausea, decreased appetite, pruritus, fever, diarrhea, rash, and arthralgia (26, 72).

##### Avelumab

Avelumab is also an IgG1 antibody that primarily targets PD-L1 and was approved by the FDA in 2017 for uroepithelial cancer (73). When combined with platinum-based drugs, Avelumab produces a sustained antitumor response in patients with advanced or present metastatic uroepithelial carcinoma (26). Patients may experience side effects such as fatigue, weakness, nausea, and infusion-related reactions (29).

#### Anti-CTLA-4 Antibodies

CTLA-4 is a surface molecule expressed by activated T cells that binds to B7.1 and B7.2 ligands expressed on B lymphocytes, dendritic cells, and macrophages (68). CTLA-4 is a co-stimulatory molecule necessary for the activation of T lymphocytes (68, 74, 75). It has been established to negatively regulate the immune system; nonetheless, the mechanism of its action is not fully understood. Given that CTLA-4 is structurally related to CD28, it has been suggested that CTLA-4 can compete with CD28 in terms of ligand binding. and another also suggested that it can directly signal all the way to the CTLA-4 cytoplasmic tail (76–78), and inhibition of CTLA-4 enhances the immune response.

##### Ipilimumab

Ipilimumab, originally developed by Bristol-Meyers Squibb as an anti-CTLA-4 monoclonal antibody for the treatment of melanoma, is also used in combination with nivolumab for the treatment of advanced kidney cancer and different types of metastatic colorectal cancer (79), with common side effects including toxicity in the dermal system, gastrointestinal tract, liver, and neurological and endocrine systems (80, 81). However, the efficacy of this drug in BC is largely unknown, warranting further studies.

##### Tremelimumab

Tremelimumab is a well-recognized humanized monoclonal antibody against CTLA-4, however, it has not been approved by the FDA for cancer treatment.

#### Chimeric Antigen Receptor Weight-Targeted T Cells

CAR-T is a novel precision-targeted therapy for the treatment of tumors. The Chimeric antigen receptor (CAR) is the core component of CAR-T, which gives T cells the ability to recognize tumor antigens in an HLA-independent manner, enabling them to recognize a broader range of target antigens than natural T-cell surface receptors (TCRs) (82). It is a highly promising immunotherapy approach that has yielded good results in clinical tumor treatment in recent years through optimization and improvement.

There are currently two FDA-approved CAR-T therapies: Kymriah (Tisagenlecleucel) and Yescarta (Axicabtagene ciloleucel). CAR-T is now predominantly used for the treatment of B-cell acute lymphoblastic leukemia (B-ALL) and diffuse large B-cell lymphoma (DLBCL) (83). Due to the complexity of BC and its location in the body, the treatment of solid tumors with CAR-T cells faces multiple obstacles, such as a harsh tumor microenvironment, on-tumor or off-tumor toxicity, and unpredictable antigen specificity (84). Notwithstanding that CAR-T is already approved to treat solid tumors such as BC, clinical trials on CAR-T cells for solid tumors are still being conducted on multiple fronts. CAR-T is also associated with serious adverse effects (85), mainly cytokine release syndrome (CRS) (86), immune effector cell-associated neurotoxic syndrome (ICANS) (87), infection, bone marrow suppression, phagocytic lymphohistiocytosis (HLH) (88), B-cell dysplasia, neurotoxicity (89), and disseminated intravascular coagulation (DIC) (90), and toxicity to other organs.

## Prevention and Treatment of Side Effects

The side effects of immune checkpoint inhibitors therapies are usually caused by the immune system attacking normal body parts in the same way it attacks cancer cells. Different types of immunotherapies can cause various side effects, many of which depend on the type of treatment, the tumor type and location, and the patient’s general health condition. Immunotherapy side effects can be mild, moderate, or even life-threatening. Some side effects can resolve on their own within a certain time frame while others persist and worsen. In such cases, it should be considered to taper the dosage, discontinue, or change the medication. Indeed, prevention of the occurrence or worsening of side effects is essential for effective treatment of these patients population. At the end of immunotherapy, it is important to observe side effects, some of which may occur months or years later (91, 92). Side effects of immune checkpoint inhibitors therapies may affect the following parts of the body. (**Table 2**)

**Table 2:** List of serious complications and brief prevention methods for bladder cancer.

Complications	Brief Prevention Methods
Infusion reaction	Strictly regulate infusion operation, closely observe patient infusion.
Sepsis	timely targeted treatment, avoid cross-infection.
Pneumonia	Improve resistance, avoid repeated infections.
Colitis	Avoid raw and cold diet, avoid repeated infections, and control with medication if necessary.
Intestinal obstruction	Medication to laxative, easy to digest diet, avoid strenuous exercise after meals.
Pancreatitis and peptic ulcer	Pay attention to dietary hygiene, inhibit gastric acid secretion, protect gastric mucosa.
Drug hepatitis	Use hepatotoxic drugs carefully, check liver function regularly, use liver protection drugs if necessary.
Renal insufficiency	Use nephrotoxic drugs carefully, check kidney function regularly.
Nephritis and urinary tract infection	Avoid holding urine, drink more water, strengthen nutrition.
Cardiac insufficiency	Absorb oxygen, control blood pressure, avoid emotional excitement, take oral vasoactive drugs if necessary.
Respiratory failure	Absorb oxygen, prevent respiratory tract infection, use ventilator if necessary.
Endocrine diseases	Pay attention to diet, strengthen exercise, use long-term maintenance medication if necessary.

General prevention: reasonable diet, pay attention to environmental hygiene, regular work and rest, avoid straining and staying up late, limit smoking and alcohol, strengthen exercise, and enhance their resistance.

When an immunotherapy drug is given to the patients through a vein, it is called an infusion. Patients receiving infusions may experience different reactions, mainly including fever, chills, accompanied by nausea, vomiting, headache and peripheral discomfort. When a mild reaction is observed, the infusion rate can be slowed down, and attention can be paid to keeping the patients warm. In case of a severe reaction, the infusion should be immediately stopped, external cooling should be provided to pyrexial patients, and anti-allergy drugs should be prescribed if necessary (93–95).

Skin problems, like rash, itching and skin photosensitivity (72), are most common in people with BC. Skin problem caused by immunotherapy are usually not serious but can be significantly uncomfortable for the patients. For rashes, corticosteroid ointments or antibiotic ointments remain the mainstay of treatment, and oral medications may be required for severe cases. For dry skin, it is recommended to use a hypoallergenic, cream-based moisturizer to prevent skin dryness, try bath products that are gentle on the skin and shower with warm water. It is essential for patients complaining of itchy skin to avoid scented skin products and use topical steroids and oral antihistamines (96). Indeed, such patients should pay attention to hydration daily, avoiding contact with allergens and exposure to sunlight (97).

Problems with the gastrointestinal tract are also some of the most common side effects related to immune checkpoint inhibitors therapies. These include colitis, diarrhea, swallowing problems, nausea and vomiting, and pain in the upper abdomen. Regular examination of abdominal signs, abdominal X-rays, abdominal ultrasound, CT, gastroscopy and enteroscopy can be used for diagnosis (98). The treatment regimen usually includes medications to inhibit hydrochloric acid and protect the digestive tract, such as proton pump inhibitors, and medications such as gastric mucosal protectors and hepatoprotective drugs can also be considered. Daily diet is carefully chosen by avoiding caffeine, alcohol, and spicy foods, eating less and more often, and regular monitoring of electrolyte levels, coupled with proper hydration and electrolyte supplementation to prevent further aggravation of the condition (99, 100).

Muscle, joint and bone problems can also occur in people who receive immune checkpoint inhibitors therapies. These can result in arthritis-type pain, swelling in joints, and muscle cramping, and even myasthenia gravis, manifest with limited range of motion and stiffness after inactivity or activity, swelling or pressure pain and redness or warmth at the joint. The diagnosis is usually made with X-rays, bone scans, CT, MRI and bone densitometry (101). Pain can be relieved with medications such as painkillers, corticosteroids, calcium tablets, vitamin D and antibiotics (102, 103). In addition, some physical therapies such as acupuncture, hot or cold compresses and massage can also be used to relieve pain (104, 105). It is worth mentioning that myasthenia gravis is a chronic autoimmune disease, the diagnosis is usually made by conducting the Tensilon test or a nerve conduction test. Acetylcholinesterase inhibitors such as neostigmine or pyridostigmine remain the mainstay of treatment of myasthenia gravis; immunosuppressive drugs such as prednisone or azathioprine also can be considered (106). Treatment with plasmapheresis and high doses of intravenous immunoglobulin may be required for cases presenting with sudden onset of symptoms (107). Adjunctive use of a ventilator may be required in cases of respiratory muscle weakness. For these problems, proper exercise, weight maintenance, and taking precautions to avoid falls are essential (102, 103).

In the urinary tract, renal inflammation and hematuria is more likely to occur in patients who with immune checkpoint inhibitors therapies compared to kidney damage and kidney failure. These can be diagnosed through complete blood count (CBC), creatinine, blood urea nitrogen, abdominal ultrasound, abdominal CT and ureteroscopy. The treatment mainly focuses on protecting kidney function, ensuring adequate rest, proper nutrition and strict control of blood pressure, blood lipids and blood sugar, coupled with management of major and minor symptoms (108–111).

The neurological side effects of immune checkpoint inhibitors therapies are mainly in the central and peripheral nervous system (112, 113), affecting your brain, senses, mind, and even movement. These are rare but can be serious side effects. A cranial CT or MRI would be a good choice for the diagnostic workup in patients with dizziness and headache combined with a history of severe illness. The treatment of neurological problems is based on neurotrophy and then, take appropriate treatment measures to deal with corresponding symptoms. For example, analgesics for headaches, anti-dizziness drugs for dizziness, etc. Although nerve damage and neurological symptoms are not preventable, most are manageable if detected early, and early treatment can also prevent symptoms from exacerbating.

Immunotherapy may cause changes in the number of blood cells and blood factors, which can lead to anemia, coagulation disorders and sepsis. It can be diagnosed with a CBC, clotting assays and blood protein tests. Anemia can be treated with blood transfusions or erythropoiesis-stimulating agents (ESAs), and a diet rich in iron, folic acid, and vitamin B12 can play a preventive role (114). Blood coagulation is a complex process involving a series of reactions involving platelets and clotting factors. Hemorrhage and thrombosis occur when the balance between clotting factors is disrupted (115). In the case of bleeding disorder, prompt supplementation of platelets, vitamin K and other pro-coagulant medications; A blood clot is a serious condition that needs treatment right away, the management of thrombosis consists of anticoagulation with warfarin or rivaroxaban, followed by thrombolytic therapy with urokinase or streptokinase. Besides, extra care should be taken during daily activities to avoid circumstances that may lead to bleeding and thrombosis (116, 117). Sepsis, on the other hand, requires the selection of appropriate antibiotics, aggressive anti-infection treatment, increasing resistance, avoiding late nights and exertion, and avoiding the intake of unclean water and food.

Immune checkpoint inhibitors therapies may cause pneumonitis, which is inflammation of the lung that can cause a cough or trouble breathing. Pneumonitis is uncommon but may be serious. Inflammatory serum biomarkers, chest X-rays, contrast-enhanced CT, and pulmonary function tests are common diagnostic methods (118). The management involves aggressive treatment with anti-inflammatory drugs, coupled with symptomatic management to relieve respiratory spasms and alleviate wheezing (119, 120). In patients with pneumonia and pleural effusion, light exercise is recommended to accelerate resorption of inflammation. It is essential for patients with pulmonary vascular thrombosis to lie down to prevent dislodging of the thrombus that can block other blood vessels. The patient should refrain from smoking and exposure to secondhand smoke. Indeed, lots of fluids are required to keep hydrated, and exposure to irritants should be avoided to avoid exacerbating the cough (120–122).

The endocrine system controls the hormones that help the body regulate many important functions, like blood pressure, blood sugar, energy, and the ability to respond to stresses like infections and injuries (14, 123). The thyroid, adrenal, pancreatic, sexual gland is a vital part of the endocrine system, and it may be triggered to become either more or less productive by immune checkpoint inhibitor treatments. The diagnosis focuses on the examination of the corresponding glands and the hormones they secrete. To treat the above endocrine side effects, the patient’s hormone levels should be assessed. If a decline is observed, treatment with hormone replacement therapy is indicated. Drugs that inhibit endocrine gland hormone release are prescribed if high levels are found. Given the insidious nature of these autoimmune events, the consequences are often ongoing and even permanent, requiring long-term hormone replacement therapy (107). Pay attention to exercise and healthy diet in daily life.

Immunotherapy may affect the heart and blood vessels. These side effects are rare but are often very serious and can be life-threatening. Includes cardiomyopathy, congestive heart failure (CHF), myocarditis, coronary artery disease, arrhythmias, heart valve damage, and pericardial disease. The clinical presentation usually consists of shortness of breath, dizziness, chest pain, edema, fatigue, etc. (124). Regular physical examinations heartbeat sounds, vascular murmurs, laboratory tests, cardiac enzyme profile, BNP, echocardiograms, chest X-rays, electrocardiograms, multi-gate acquisition scans (MUGA), cardiac MRI and angiograms can be used to diagnose heart problems. The treatment plan usually consists of cardio-protective drugs such as dexrazoxane (Zinecard) which help prevent cardiac problems induced by anthracyclines. Inotropes (digitalis), diuretics and hypertensive drugs should also be considered (125–128). Take care to avoid emotional excitement in daily life and have a light diet is necessary.

## Conclusion

Immunotherapy is regarded as a promising and more effective therapeutic measure in the treatment of various types of cancer. However, the side effects of it are underestimated currently. The unpredictable occurrence of serious side effects not only causes serious health damage to patients, but also increases the medical burden to some extent. The clinical management of side effects in patients today is mainly empirical. Therefore, a uniform and systematic guideline to control the side effects of immunotherapy is necessary. Based on the insufficiency understanding on the side effects of immunotherapy, more follow-up data on the side effect is needed, as well as prospective, multicenter, large-scale trials on the preventive measures. Above all, further research on the molecular mechanisms and clinical efficacy of the side effects of immunotherapy is still urgent.

## Prospect

Although immunotherapy developed and achieved widespread application in clinical cancer therapy, further research is necessary in immunotherapy for bladder cancer, especially in the systemic response, which may exert role in the development of side effects of immunotherapy. Furthermore, the genetic diversity of bladder cancer and epigenetic modification are also relevant to efficacy and side effects.

During recent years, researchers proposed new methods to improve the efficacy of immunotherapy and mitigate side effects, such as activation of thioredoxin, bacterial colony transplantation, and ferroptosis induction. At the same time, alternative therapeutic measures are beneficial in alleviating the symptoms of drug complications, such as rehabilitation therapy, Chinese acupuncture, and physiotherapy. In addition, combination or individualized treatments depending on the diversity of the patient is also a good choice. Hence, develop new methods to alleviate side effect would be an important subject in immunotherapy.

## Author Contributions

KL and SF searched for literature and wrote the first draft of this article. XZ edited tables and figures. JZ and GZ reviewed the manuscript and polished the grammar. All authors contributed to the article and approved the submitted version.

## Funding

This work was supported by the National Natural Science Foundation of China (No. 81860456); The Jiangxi Natural Science Foundation (No. 20202BABL206031).

## Conflict of Interest

The authors declare that the research was conducted in the absence of any commercial or financial relationships that could be construed as a potential conflict of interest.

## Publisher’s Note

All claims expressed in this article are solely those of the authors and do not necessarily represent those of their affiliated organizations, or those of the publisher, the editors and the reviewers. Any product that may be evaluated in this article, or claim that may be made by its manufacturer, is not guaranteed or endorsed by the publisher.

## References

[1] Ferlay J. Global Cancer Observatory: Cancer Today (2018). Available at: https://gco.iarc.fr/today (Accessed date 01 May 2019).

[2] Brahmer J Reckamp KL Baas P Crinò L Eberhardt WE Poddubskaya E. Nivolumab Versus Docetaxel in Advanced Squamous-Cell Non-Small-Cell Lung Cancer. N Engl J Med (2015) 373(2):123–35. doi: 10.1056/NEJMoa1504627 PMC4681400 26028407

[3] Richters A Aben KKH Kiemeney L. The Global Burden of Urinary Bladder Cancer: An Update. World J Urol (2020) 38(8):1895–904. doi: 10.1007/s00345-019-02984-4 PMC7363726 31676912

[4] Kim HS Seo HK. Immune Checkpoint Inhibitors for Urothelial Carcinoma. Investig Clin Urol (2018) 59(5):285–96. doi: 10.4111/icu.2018.59.5.285 PMC6121021 30182073

[5] Saginala K Barsouk A Aluru JS Rawla P Padala SA Barsouk A. Epidemiology of Bladder Cancer. Med Sci (Basel) (2020) 8(1):5–15. doi: 10.3390/medsci8010015 PMC7151633 32183076

[6] Chien TM Chan TC Huang SK Yeh BW Li WM Huang CN. Role of Microtubule-Associated Protein 1b in Urothelial Carcinoma: Overexpression Predicts Poor Prognosis. Cancers (Basel) (2020) 12(3):630. doi: 10.3390/cancers12030630 PMC7139768 32182788

[7] AkgÜL A Ahmed N Raza A Iqbal Z Rafiq M Rehman MA. A Fractal Fractional Model for Cervical Cancer Due to Human Papillomavirus Infection. Fractals (2021) 29(05):2140015. doi: 10.1142/S0218348X21400156

[8] Jagodinsky JC Harari PM Morris ZS. The Promise of Combining Radiation Therapy With Immunotherapy. Int J Radiat Oncol Biol Phys (2020) 108(1):6–16. doi: 10.1016/j.ijrobp.2020.04.023 32335187 PMC7442714

[9] Fyfe GA Fisher RI Rosenberg SA Sznol M Parkinson DR Louie AC. Long-Term Response Data for 255 Patients With Metastatic Renal Cell Carcinoma Treated With High-Dose Recombinant Interleukin-2 Therapy. J Clin Oncol (1996) 14(8):2410–1. doi: 10.1200/JCO.1996.14.8.2410 8708739

[10] Atkins MB Lotze MT Dutcher JP Fisher RI Weiss G Margolin K. High-Dose Recombinant Interleukin 2 Therapy for Patients With Metastatic Melanoma: Analysis of 270 Patients Treated Between 1985 and 1993. J Clin Oncol (1999) 17(7):2105–16. doi: 10.1200/JCO.1999.17.7.2105 10561265

[11] Havel JJ Chowell D Chan TA. The Evolving Landscape of Biomarkers for Checkpoint Inhibitor Immunotherapy. Nat Rev Cancer (2019) 19(3):133–50. doi: 10.1038/s41568-019-0116-x PMC6705396 30755690

[12] Weber JS Yang JC Atkins MB Disis ML. Toxicities of Immunotherapy for the Practitioner. J Clin Oncol (2015) 33(18):2092–9. doi: 10.1200/JCO.2014.60.0379 PMC4881375 25918278

[13] Marrone KA Ying W Naidoo J. Immune-Related Adverse Events From Immune Checkpoint Inhibitors. Clin Pharmacol Ther (2016) 100(3):242–51. doi: 10.1002/cpt.394 27170616

[14] Michot JM Bigenwald C Champiat S Collins M Carbonnel F Postel-Vinay S. Immune-Related Adverse Events With Immune Checkpoint Blockade: A Comprehensive Review. Eur J Cancer (2016) 54:139–48. doi: 10.1016/j.ejca.2015.11.016 26765102

[15] Pettenati C Ingersoll MA. Mechanisms of BCG Immunotherapy and its Outlook for Bladder Cancer. Nat Rev Urol (2018) 15(10):615–25. doi: 10.1038/s41585-018-0055-4 29991725

[16] Jurkowska K Długosz A. Research on New Drugs in the Therapy of Bladder Cancer (BC). Postępy Hig Med Dosw (2018) 72:442–8. doi: 10.5604/01.3001.0012.0539

[17] Eltze E Wülfing C Von Struensee D Piechota H Buerger H Hertle L. Cox-2 and Her2/neu Co-Expression in Invasive Bladder Cancer. Int J Oncol (2005) 26(6):1525–31. doi: 10.3892/ijo.26.6.1525 15870865

[18] Dhawan D Jeffreys AB Zheng R Stewart JC Knapp DW. Cyclooxygenase-2 Dependent and Independent Antitumor Effects Induced by Celecoxib in Urinary Bladder Cancer Cells. Mol Cancer Ther (2008) 7(4):897–904. doi: 10.1158/1535-7163.MCT-07-0313 18413803

[19] Sharma P Callahan MK Bono P Kim J Spiliopoulou P Calvo E. Nivolumab Monotherapy in Recurrent Metastatic Urothelial Carcinoma (CheckMate 032): A Multicentre, Open-Label, Two-Stage, Multi-Arm, Phase 1/2 Trial. Lancet Oncol (2016) 17(11):1590–8. doi: 10.1016/S1470-2045(16)30496-X PMC5648054 27733243

[20] Sharma P Retz M Siefker-Radtke A Baron A Necchi A Bedke J. Nivolumab in Metastatic Urothelial Carcinoma After Platinum Therapy (CheckMate 275): A Multicentre, Single-Arm, Phase 2 Trial. Lancet Oncol (2017) 18(3):312–22. doi: 10.1016/S1470-2045(17)30065-7 28131785

[21] Bellmunt J de Wit R Vaughn DJ Fradet Y Lee JL Fong L. Pembrolizumab as Second-Line Therapy for Advanced Urothelial Carcinoma. N Engl J Med (2017) 376(11):1015–26. doi: 10.1056/NEJMoa1613683 PMC5635424 28212060

[22] Balar AV Castellano D O'Donnell PH Grivas P Vuky J Powles T. First-Line Pembrolizumab in Cisplatin-Ineligible Patients With Locally Advanced and Unresectable or Metastatic Urothelial Cancer (KEYNOTE-052): A Multicentre, Single-Arm, Phase 2 Study. Lancet Oncol (2017) 18(11):1483–92. doi: 10.1016/S1470-2045(17)30616-2 28967485

[23] Plimack ER Bellmunt J Gupta S Berger R Chow LQ Juco J. Safety and Activity of Pembrolizumab in Patients With Locally Advanced or Metastatic Urothelial Cancer (KEYNOTE-012): A Non-Randomised, Open-Label, Phase 1b Study. Lancet Oncol (2017) 18(2):212–20. doi: 10.1016/S1470-2045(17)30007-4 28081914

[24] Massard C Gordon MS Sharma S Rafii S Wainberg ZA Luke J. Safety and Efficacy of Durvalumab (MEDI4736), an Anti-Programmed Cell Death Ligand-1 Immune Checkpoint Inhibitor, in Patients With Advanced Urothelial Bladder Cancer. J Clin Oncol (2016) 34(26):3119–25. doi: 10.1200/JCO.2016.67.9761 PMC5569690 27269937

[25] Powles T O'Donnell PH Massard C Arkenau HT Friedlander TW Hoimes CJ. Efficacy and Safety of Durvalumab in Locally Advanced or Metastatic Urothelial Carcinoma: Updated Results From a Phase 1/2 Open-Label Study. JAMA Oncol (2017) 3(9):e172411. doi: 10.1001/jamaoncol.2017.2411 28817753 PMC5824288

[26] Rosenberg JE Hoffman-Censits J Powles T van der Heijden MS Balar AV Necchi A. Atezolizumab in Patients With Locally Advanced and Metastatic Urothelial Carcinoma Who Have Progressed Following Treatment With Platinum-Based Chemotherapy: A Single-Arm, Multicentre, Phase 2 Trial. Lancet (2016) 387(10031):1909–20. doi: 10.1016/S0140-6736(16)00561-4 PMC5480242 26952546

[27] Powles T Durán I van der Heijden MS Loriot Y Vogelzang NJ De Giorgi U. Atezolizumab Versus Chemotherapy in Patients With Platinum-Treated Locally Advanced or Metastatic Urothelial Carcinoma (IMvigor211): A Multicentre, Open-Label, Phase 3 Randomised Controlled Trial. Lancet (2018) 391(10122):748–57. doi: 10.1016/S0140-6736(17)33297-X 29268948

[28] Eckstein M Erben P Kriegmair MC Worst TS Weiß CA Wirtz RM. Performance of the Food and Drug Administration/EMA-Approved Programmed Cell Death Ligand-1 Assays in Urothelial Carcinoma With Emphasis on Therapy Stratification for First-Line Use of Atezolizumab and Pembrolizumab. Eur J Cancer (2019) 106:234–43. doi: 10.1016/j.ejca.2018.11.007 30528808

[29] Apolo AB Infante JR Balmanoukian A Patel MR Wang D Kelly K. Avelumab, an Anti-Programmed Death-Ligand 1 Antibody, In Patients With Refractory Metastatic Urothelial Carcinoma: Results From a Multicenter, Phase Ib Study. J Clin Oncol (2017) 35(19):2117–24. doi: 10.1200/JCO.2016.71.6795 PMC5493051 28375787

[30] Patel MR Ellerton J Infante JR Agrawal M Gordon M Aljumaily R. Avelumab in Metastatic Urothelial Carcinoma After Platinum Failure (JAVELIN Solid Tumor): Pooled Results From Two Expansion Cohorts of an Open-Label, Phase 1 Trial. Lancet Oncol (2018) 19(1):51–64. doi: 10.1016/S1470-2045(17)30900-2 29217288 PMC7984727

[31] Powles T Park SH Voog E Caserta C Valderrama BP Gurney H. Avelumab Maintenance Therapy for Advanced or Metastatic Urothelial Carcinoma. N Engl J Med (2020) 383(13):1218–30. doi: 10.1056/NEJMoa2002788 32945632

[32] Crispen PL Kusmartsev S. Mechanisms of Immune Evasion in Bladder Cancer. Cancer Immunol Immunother (2020) 69(1):3–14. doi: 10.1007/s00262-019-02443-4 31811337 PMC6949323

[33] Farman M Akgül A Ahmad A Imtiaz S. Analysis and Dynamical Behavior of Fractional-Order Cancer Model With Vaccine Strategy. Math Methods Appl Sci (2020) 43(7):4871–82. doi: 10.1002/mma.6240

[34] Durek C Brandau S Ulmer AJ Flad HD Jocham D Böhle A. Bacillus-Calmette-Guérin (BCG) and 3D Tumors: An in Vitro Model for the Study of Adhesion and Invasion. J Urol (1999) 162(2):600–5. doi: 10.1016/s0022-5347(05)68633-8 10411094

[35] De Boer EC De Jong WH Steerenberg PA Aarden LA Tetteroo E De Groot ER. Induction of Urinary Interleukin-1 (IL-1), IL-2, IL-6, and Tumour Necrosis Factor During Intravesical Immunotherapy With Bacillus Calmette-Guérin in Superficial Bladder Cancer. Cancer Immunol Immunother (1992) 34(5):306–12. doi: 10.1007/BF01741551 PMC11038144 1540977

[36] Luo Y Chen X O'Donnell MA. Mycobacterium Bovis Bacillus Calmette-Guérin (BCG) Induces Human CC- and CXC-Chemokines In Vitro and *In Vivo* . Clin Exp Immunol (2007) 147(2):370–8. doi: 10.1111/j.1365-2249.2006.03288.x PMC1810474 17223980

[37] Kawai K Miyazaki J Joraku A Nishiyama H Akaza H. Bacillus Calmette-Guerin (BCG) Immunotherapy for Bladder Cancer: Current Understanding and Perspectives on Engineered BCG Vaccine. Cancer Sci (2013) 104(1):22–7. doi: 10.1111/cas.12075 PMC7657210 23181987

[38] Prescott S James K Hargreave TB Chisholm GD Smyth JF. Intravesical Evans Strain BCG Therapy: Quantitative Immunohistochemical Analysis of the Immune Response Within the Bladder Wall. J Urol (1992) 147(6):1636–42. doi: 10.1016/S0022-5347(17)37668-1 1593713

[39] Ratliff TL Ritchey JK Yuan JJ Andriole GL Catalona WJ. T-Cell Subsets Required for Intravesical BCG Immunotherapy for Bladder Cancer. J Urol (1993) 150(3):1018–23. doi: 10.1016/S0022-5347(17)35678-1 8102183

[40] Brandau S Riemensberger J Jacobsen M Kemp D Zhao W Zhao X. NK Cells Are Essential for Effective BCG Immunotherapy. Int J Cancer (2001) 92(5):697–702. doi: 10.1002/1097-0215(20010601)92:5<697::AID-IJC1245>3.0.CO;2-Z 11340575

[41] Suttmann H Riemensberger J Bentien G Schmaltz D Stöckle M Jocham D. Neutrophil Granulocytes are Required for Effective Bacillus Calmette-Guérin Immunotherapy of Bladder Cancer and Orchestrate Local Immune Responses. Cancer Res (2006) 66(16):8250–7. doi: 10.1158/0008-5472.CAN-06-1416 16912205

[42] Pryor K Goddard J Goldstein D Stricker P Russell P Golovsky D. Bacillus Calmette-Guerin (BCG) Enhances Monocyte- and Lymphocyte-Mediated Bladder Tumour Cell Killing. Br J Cancer (1995) 71(4):801–7. doi: 10.1038/bjc.1995.155 PMC2033737 7710947

[43] De Boer EC De Jong WH van der Meijden AP Steerenberg PA Witjes JA Vegt PD. Presence of Activated Lymphocytes in the Urine of Patients With Superficial Bladder Cancer After Intravesical Immunotherapy With Bacillus Calmette-Guérin. Cancer Immunol Immunother (1991) 33(6):411–6. doi: 10.1007/BF01741603 PMC11038680 1878894

[44] Chen F Zhang G Iwamoto Y See WA. BCG Directly Induces Cell Cycle Arrest in Human Transitional Carcinoma Cell Lines as a Consequence of Integrin Cross-Linking. BMC Urol (2005) 5:8. doi: 10.1186/1471-2490-5-8 15890073 PMC1174876

[45] Zhang C Berndt-Paetz M Neuhaus J. Identification of Key Biomarkers in Bladder Cancer: Evidence From a Bioinformatics Analysis. Diagn (Basel) (2020) 10(2):66. doi: 10.3390/diagnostics10020066 PMC7168923 31991631

[46] Lockyer CR Gillatt DA. BCG Immunotherapy for Superficial Bladder Cancer. J R Soc Med (2001) 94(3):119–23. doi: 10.1177/014107680109400305 PMC1297926 11285791

[47] Akgül A Farman M Ahmad A Saleem MU. Bacillus Calmette Guerin (BCG) Immunotherapy for Bladder Cancer: A Control and Mathematical Analysis. Int J Appl Comput Math (2021) 7(6):254. doi: 10.1007/s40819-021-01191-3

[48] Jurkowska K Długosz A. Research on New Drugs in the Therapy of Bladder Cancer (BC) Postepy Hig. Med Dosw (2018) 72:442–8. doi: 10.5604/01.3001.0012.0539

[49] Pinto-Leite R Arantes-Rodrigues R Sousa N Oliveira PA Santos L. mTOR Inhibitors in Urinary Bladder Cancer. Tumour Biol (2016) 37(9):11541–51. doi: 10.1007/s13277-016-5083-1 27235118

[50] Everolimus (Afinitor). Available at: https://www.fda.gov/drugs/resources-information-approved-drugs/everolimus-afinitor (Accessed February 26).

[51] Liu X Wu Y Zhou Z Huang M Deng W Wang Y. Celecoxib Inhibits the Epithelial-to-Mesenchymal Transition in Bladder Cancer Via the miRNA-145/TGFBR2/Smad3 Axis. Int J Mol Med (2019) 44(2):683–93. doi: 10.3892/ijmm.2019.4241 PMC6605707 31198976

[52] Dunn GP Bruce AT Ikeda H Old LJ Schreiber RD. Cancer Immunoediting: From Immunosurveillance to Tumor Escape. Nat Immunol (2002) 3(11):991–8. doi: 10.1038/ni1102-991 12407406

[53] Good-Jacobson KL Szumilas CG Chen L Sharpe AH Tomayko MM Shlomchik MJ. PD-1 Regulates Germinal Center B Cell Survival and the Formation and Affinity of Long-Lived Plasma Cells. Nat Immunol (2010) 11(6):535–42. doi: 10.1038/ni.1877 PMC2874069 20453843

[54] Iwama S De Remigis A Callahan MK Slovin SF Wolchok JD Caturegli P. Pituitary Expression of CTLA-4 Mediates Hypophysitis Secondary to Administration of CTLA-4 Blocking Antibody. Sci Transl Med (2014) 6(230):230ra45. doi: 10.1126/scitranslmed.3008002 24695685

[55] Zitvogel L Kroemer G. Targeting PD-1/PD-L1 Interactions for Cancer Immunotherapy. Oncoimmunology (2012) 1(8):1223–5. doi: 10.4161/onci.21335 PMC3518493 23243584

[56] Naidoo J Page DB Li BT Connell LC Schindler K Lacouture ME. Toxicities of the Anti-PD-1 and Anti-PD-L1 Immune Checkpoint Antibodies. Ann Oncol (2015) 26(12):2375–91. doi: 10.1093/annonc/mdv383 PMC6267867 26371282

[57] Callahan MK Wolchok JD. At the Bedside: CTLA-4- and PD-1-Blocking Antibodies in Cancer Immunotherapy. J Leukoc Biol (2013) 94(1):41–53. doi: 10.1189/jlb.1212631 23667165 PMC4051187

[58] Weber JS Postow M Lao CD Schadendorf D. Management of Adverse Events Following Treatment With Anti-Programmed Death-1 Agents. Oncologist (2016) 21(10):1230–40. doi: 10.1634/theoncologist.2016-0055 PMC5061539 27401894

[59] Rundo F Spampinato C Banna GL Conoci S. Advanced Deep Learning Embedded Motion Radiomics Pipeline for Predicting Anti-PD-1/PD-L1 Immunotherapy Response in the Treatment of Bladder Cancer: Preliminary Results. Electronics (2019) 8:1134. doi: 10.3390/electronics8101134

[60] Alsaab HO Sau S Alzhrani R Tatiparti K Bhise K Kashaw SK. PD-1 and PD-L1 Checkpoint Signaling Inhibition for Cancer Immunotherapy: Mechanism, Combinations, and Clinical Outcome. Front Pharmacol (2017) 8:561. doi: 10.3389/fphar.2017.00561 28878676 PMC5572324

[61] Bristol-Myers Squibb Receives FDA Approval for Opdivo (Nivolumab) in Previously Treated Locally Advanced or Metastatic Urothelial Carcinoma Available at: https://www.drugs.com/newdrugs/bristol-myers-squibb-receives-fda-approval-opdivo-nivolumab-previously-treated-locally-advanced-4484.html (Accessed on 20 January 2020).

[62] Jain RK Snyders T Nandagopal L Garje R Zakharia Y Gupta S. Immunotherapy Advances in Urothelial Carcinoma. Curr Treat Opt Oncol (2018) 19(12):79. doi: 10.1007/s11864-018-0598-x 30554335

[63] Weber JS Hodi FS Wolchok JD Topalian SL Schadendorf D Larkin J. Safety Profile of Nivolumab Monotherapy: A Pooled Analysis of Patients With Advanced Melanoma. J Clin Oncol (2017) 35(7):785–92. doi: 10.1200/JCO.2015.66.1389 28068177

[64] Larkin J Lao CD Urba WJ McDermott DF Horak C Jiang J. Efficacy and Safety of Nivolumab in Patients With BRAF V600 Mutant and BRAF Wild-Type Advanced Melanoma: A Pooled Analysis of 4 Clinical Trials. JAMA Oncol (2015) 1(4):433–40. doi: 10.1001/jamaoncol.2015.1184 26181250

[65] Patterson K Prabhu V Xu R Li H Meng Y Zarabi N. Cost-Effectiveness of Pembrolizumab for Patients With Advanced, Unresectable, or Metastatic Urothelial Cancer Ineligible for Cisplatin-Based Therapy. Eur Urol Oncol (2019) 2(5):565–71. doi: 10.1016/j.euo.2018.09.009 31412011

[66] Morsch R Rose M Maurer A Cassataro MA Braunschweig T Knüchel R. Therapeutic Implications of PD-L1 Expression in Bladder Cancer With Squamous Differentiation. BMC Cancer (2020) 20(1):230. doi: 10.1186/s12885-020-06727-2 32188412 PMC7079494

[67] Mancuso JG Foulkes WD Pollak MN. Cancer Immunoprevention: A Case Report Raising the Possibility of "Immuno-Interception". Cancer Prev Res (Phila) (2020) 13(4):351–6. doi: 10.1158/1940-6207.CAPR-19-0528 32241906

[68] Farina MS Lundgren KT Bellmunt J. Immunotherapy in Urothelial Cancer: Recent Results and Future Perspectives. Drugs (2017) 77(10):1077–89. doi: 10.1007/s40265-017-0748-7 28493171

[69] Zajac M Boothman AM Ben Y Gupta A Jin X Mistry A. Analytical Validation and Clinical Utility of an Immunohistochemical Programmed Death Ligand-1 Diagnostic Assay and Combined Tumor and Immune Cell Scoring Algorithm for Durvalumab in Urothelial Carcinoma. Arch Pathol Lab Med (2019) 143(6):722–31. doi: 10.5858/arpa.2017-0555-OA 30457897

[70] Powles T Eder JP Fine GD Braiteh FS Loriot Y Cruz C. MPDL3280A (Anti-PD-L1) Treatment Leads to Clinical Activity in Metastatic Bladder Cancer. Nature (2014) 515(7528):558–62. doi: 10.1038/nature13904 25428503

[71] FDA Atezolizumab for Urothelial Carcinoma. Available at: https://www.fda.gov/drugs/resources-information-approved-drugs/atezolizumab-urothelial-carcinoma (Accessed on 10 December 2019).

[72] Teulings HE Limpens J Jansen SN Zwinderman AH Reitsma JB Spuls PI. Vitiligo-Like Depigmentation in Patients With Stage III-IV Melanoma Receiving Immunotherapy and its Association With Survival: A Systematic Review and Meta-Analysis. J Clin Oncol (2015) 33(7):773–81. doi: 10.1200/JCO.2014.57.4756 25605840

[73] FDA FDA Approves Bavencio (Avelumab) for Metastatic Merkel Cell Carcinoma. Available at: https://www.drugs.com/newdrugs/fda-approves-bavencio-avelumab-metastatic-merkel-cell-carcinoma-4502.html (Accessed on 10 December 2019).

[74] Hojeij R Domingos-Pereira S Nkosi M Gharbi D Derré L Schiller JT. Immunogenic Human Papillomavirus Pseudovirus-Mediated Suicide-Gene Therapy for Bladder Cancer. Int J Mol Sci (2016) 17(7):1125. doi: 10.3390/ijms17071125 PMC4964499 27428950

[75] Schulz WA Sørensen KD. Epigenetics of Urological Cancers. Int J Mol Sci (2019) 20(19):4775. doi: 10.3390/ijms20194775 PMC6802188 31561442

[76] Peggs KS Quezada SA Chambers CA Korman AJ Allison JP. Blockade of CTLA-4 on Both Effector and Regulatory T Cell Compartments Contributes to the Antitumor Activity of Anti-CTLA-4 Antibodies. J Exp Med (2009) 206(8):1717–25. doi: 10.1084/jem.20082492 PMC2722174 19581407

[77] van der Merwe PA Davis SJ. Molecular Interactions Mediating T Cell Antigen Recognition. Annu Rev Immunol (2003) 21:659–84. doi: 10.1146/annurev.immunol.21.120601.141036 12615890

[78] Carreno BM Bennett F Chau TA Ling V Luxenberg D Jussif J. CTLA-4 (CD152) can Inhibit T Cell Activation by Two Different Mechanisms Depending on its Level of Cell Surface Expression. J Immunol (2000) 165(3):1352–6. doi: 10.4049/jimmunol.165.3.1352 10903737

[79] Yervoy Approval History. Available at: https://www.drugs.com/history/yervoy.html (Accessed on 22 January 2020).

[80] Weber JS Kähler KC Hauschild A. Management of Immune-Related Adverse Events and Kinetics of Response With Ipilimumab. J Clin Oncol (2012) 30(21):2691–7. doi: 10.1200/JCO.2012.41.6750 22614989

[81] Bot I Blank CU Boogerd W Brandsma D. Neurological Immune-Related Adverse Events of Ipilimumab. Pract Neurol (2013) 13(4):278–80. doi: 10.1136/practneurol-2012-000447 23487828

[82] Sadelain M Rivière I Brentjens R. Targeting Tumours With Genetically Enhanced T Lymphocytes. Nat Rev Cancer (2003) 3(1):35–45. doi: 10.1038/nrc971 12509765

[83] Fournier C Martin F Zitvogel L Kroemer G Galluzzi L Apetoh L. Trial Watch: Adoptively Transferred Cells for Anticancer Immunotherapy. Oncoimmunology (2017) 6(11):e1363139. doi: 10.1080/2162402X.2017.1363139 29147628 PMC5674950

[84] Zhang H Ye ZL Yuan ZG Luo ZQ Jin HJ Qian QJ. New Strategies for the Treatment of Solid Tumors With CAR-T Cells. Int J Biol Sci (2016) 12(6):718–29. doi: 10.7150/ijbs.14405 PMC4870715 27194949

[85] Brudno JN Kochenderfer JN. Toxicities of Chimeric Antigen Receptor T Cells: Recognition and Management. Blood (2016) 127(26):3321–30. doi: 10.1182/blood-2016-04-703751 PMC4929924 27207799

[86] Kochenderfer JN Dudley ME Feldman SA Wilson WH Spaner DE Maric I. B-Cell Depletion and Remissions of Malignancy Along With Cytokine-Associated Toxicity in a Clinical Trial of Anti-CD19 Chimeric-Antigen-Receptor-Transduced T Cells. Blood (2012) 119(12):2709–20. doi: 10.1182/blood-2011-10-384388 PMC3327450 22160384

[87] Locke FL Neelapu SS Bartlett NL Siddiqi T Chavez JC Hosing CM. Phase 1 Results of ZUMA-1: A Multicenter Study of KTE-C19 Anti-CD19 CAR T Cell Therapy in Refractory Aggressive Lymphoma. Mol Ther (2017) 25(1):285–95. doi: 10.1016/j.ymthe.2016.10.020 PMC5363293 28129122

[88] Neelapu SS Locke FL Bartlett NL Lekakis LJ Miklos DB Jacobson CA. Axicabtagene Ciloleucel CAR T-Cell Therapy in Refractory Large B-Cell Lymphoma. N Engl J Med (2017) 377(26):2531–44. doi: 10.1056/NEJMoa1707447 PMC5882485 29226797

[89] Turtle CJ Hanafi LA Berger C Gooley TA Cherian S Hudecek M. CD19 CAR-T Cells of Defined CD4+:CD8+ Composition in Adult B Cell ALL Patients. J Clin Invest (2016) 126(6):2123–38. doi: 10.1172/JCI85309 PMC4887159 27111235

[90] Davila ML Riviere I Wang X Bartido S Park J Curran K. Efficacy and Toxicity Management of 19-28z CAR T Cell Therapy in B Cell Acute Lymphoblastic Leukemia. Sci Transl Med (2014) 6(224):224ra25. doi: 10.1126/scitranslmed.3008226 PMC4684949 24553386

[91] Day D Hansen AR. Immune-Related Adverse Events Associated With Immune Checkpoint Inhibitors. BioDrugs (2016) 30(6):571–84. doi: 10.1007/s40259-016-0204-3 27848165

[92] Boutros C Tarhini A Routier E Lambotte O Ladurie FL Carbonnel F. Safety Profiles of Anti-CTLA-4 and Anti-PD-1 Antibodies Alone and in Combination. Nat Rev Clin Oncol (2016) 13(8):473–86. doi: 10.1038/nrclinonc.2016.58 27141885

[93] Lenz HJ. Management and Preparedness for Infusion and Hypersensitivity Reactions. Oncologist (2007) 12(5):601–9. doi: 10.1634/theoncologist.12-5-601 17522249

[94] Comer H Cardwell K. Brentuximab Vedotin Infusion Reaction Management: A Case Study. J Adv Pract Oncol (2017) 8(6):626–9. PMC6167082 30310723

[95] Roselló S Blasco I García Fabregat L Cervantes A Jordan K. Management of Infusion Reactions to Systemic Anticancer Therapy: ESMO Clinical Practice Guidelines. Ann Oncol (2017) 28(suppl_4):iv100–18. doi: 10.1093/annonc/mdx216 28881914

[96] Muntyanu A Netchiporouk E Gerstein W Gniadecki R Litvinov IV. Cutaneous Immune-Related Adverse Events (irAEs) to Immune Checkpoint Inhibitors: A Dermatology Perspective on Management [Formula: See Text]. J Cutan Med Surg (2021) 25(1):59–76. doi: 10.1177/1203475420943260 32746624

[97] Soliman YS Hashim PW Farberg AS Goldenberg G. The Role of Diet in Preventing Photoaging and Treating Common Skin Conditions. Cutis (2019) 103(3):153–6. 31039233

[98] Jackson P Vigiola Cruz M. Intestinal Obstruction: Evaluation and Management. Am Fam Phys (2018) 98(6):362–7. 30215917

[99] Whelan K Schneider SM. Mechanisms, Prevention, and Management of Diarrhea in Enteral Nutrition. Curr Opin Gastroenterol (2011) 27(2):152–9. doi: 10.1097/MOG.0b013e32834353cb 21191288

[100] Prichard DO Bharucha AE. Recent Advances in Understanding and Managing Chronic Constipation. F1000Res (2018) 7:1640. doi: 10.12688/f1000research.15900.1 PMC6192438 30364088

[101] Lane JM Russell L Khan SN. Osteoporosis. Clin Orthop Relat Res (2000) 372):139–50. doi: 10.1097/00003086-200003000-00016 10738423

[102] Spain L Diem S Larkin J. Management of Toxicities of Immune Checkpoint Inhibitors. Cancer Treat Rev (2016) 44:51–60. doi: 10.1016/j.ctrv.2016.02.001 26874776

[103] Miller PD. Management of Severe Osteoporosis. Expert Opin Pharmacother (2016) 17(4):473–88. doi: 10.1517/14656566.2016.1124856 26605922

[104] Zimmer L Goldinger SM Hofmann L Loquai C Ugurel S Thomas I. Neurological, Respiratory, Musculoskeletal, Cardiac and Ocular Side-Effects of Anti-PD-1 Therapy. Eur J Cancer (2016) 60:210–25. doi: 10.1016/j.ejca.2016.02.024 27084345

[105] Cohen SP Raja SN. Pathogenesis, Diagnosis, and Treatment of Lumbar Zygapophysial (Facet) Joint Pain. Anesthesiology (2007) 106(3):591–614. doi: 10.1097/00000542-200703000-00024 17325518

[106] Makarious D Horwood K Coward JIG. Myasthenia Gravis: An Emerging Toxicity of Immune Checkpoint Inhibitors. Eur J Cancer (2017) 82:128–36. doi: 10.1016/j.ejca.2017.05.041 28666240

[107] Haanen J Carbonnel F Robert C Kerr KM Peters S Larkin J. Management of Toxicities From Immunotherapy: ESMO Clinical Practice Guidelines for Diagnosis, Treatment and Follow-Up. Ann Oncol (2017) 28(suppl_4):iv119–42. doi: 10.1093/annonc/mdx225 28881921

[108] Foxman B. Urinary Tract Infection Syndromes: Occurrence, Recurrence, Bacteriology, Risk Factors, and Disease Burden. Infect Dis Clin North Am (2014) 28(1):1–13. doi: 10.1016/j.idc.2013.09.003 24484571

[109] Avellino GJ Bose S Wang DS. Diagnosis and Management of Hematuria. Surg Clin North Am (2016) 96(3):503–15. doi: 10.1016/j.suc.2016.02.007 27261791

[110] Stevens PE Levin A. Evaluation and Management of Chronic Kidney Disease: Synopsis of the Kidney Disease: Improving Global Outcomes 2012 Clinical Practice Guideline. Ann Intern Med (2013) 158(11):825–30. doi: 10.7326/0003-4819-158-11-201306040-00007 23732715

[111] Chenoweth CE Gould CV Saint S. Diagnosis, Management, and Prevention of Catheter-Associated Urinary Tract Infections. Infect Dis Clin North Am (2014) 28(1):105–19. doi: 10.1016/j.idc.2013.09.002 PMC9580547 24484578

[112] Wick W Hertenstein A Platten M. Neurological Sequelae of Cancer Immunotherapies and Targeted Therapies. Lancet Oncol (2016) 17(12):e529–41. doi: 10.1016/S1470-2045(16)30571-X 27924751

[113] Wang ML Rivlin M Graham JG Beredjiklian PK. Peripheral Nerve Injury, Scarring, and Recovery. Connect Tissue Res (2019) 60(1):3–9. doi: 10.1080/03008207.2018.1489381 30187777

[114] Dan K. [Drug-Induced Anemia]. Nihon Rinsho (2008) 66(3):540–3. 18326323

[115] Lisman T Intagliata NM. Bleeding and Thrombosis in Patients With Liver Diseases. Semin Thromb Hemost (2020) 46(6):653–5. doi: 10.1055/s-0040-1715453 32932541

[116] Johnstone C Rich SE. Bleeding in Cancer Patients and its Treatment: A Review. Ann Palliat Med (2018) 7(2):265–73. doi: 10.21037/apm.2017.11.01 29307210

[117] Moik F Ay C. How I Manage Cancer-Associated Thrombosis. Hamostaseologie (2020) 40(1):38–46. doi: 10.1055/s-0039-3402806 31986545

[118] Jany B Welte T. Pleural Effusion in Adults-Etiology, Diagnosis, and Treatment. Dtsch Arztebl Int (2019) 116(21):377–86. doi: 10.3238/arztebl.2019.0377 PMC6647819 31315808

[119] Skřičková J. [Pneumonia in Immunocompromised Persons]. Vnitr Lek (2018) 63(11):786–95. 29303280

[120] Beaudoin S Gonzalez AV. Evaluation of the Patient With Pleural Effusion. Cmaj (2018) 190(10):E291–e295. doi: 10.1503/cmaj.170420 29530870 PMC5849448

[121] Stein PD. Acute Pulmonary Embolism. Dis Mon (1994) 40(9):467–523. 8076500

[122] Perelas A Silver RM Arrossi AV Highland KB. Systemic Sclerosis-Associated Interstitial Lung Disease. Lancet Respir Med (2020) 8(3):304–20. doi: 10.1016/S2213-2600(19)30480-1 32113575

[123] Sznol M Postow MA Davies MJ Pavlick AC Plimack ER Shaheen M. Endocrine-Related Adverse Events Associated With Immune Checkpoint Blockade and Expert Insights on Their Management. Cancer Treat Rev (2017) 58:70–6. doi: 10.1016/j.ctrv.2017.06.002 28689073

[124] Varricchi G Marone G Mercurio V Galdiero MR Bonaduce D Tocchetti CG. Immune Checkpoint Inhibitors and Cardiac Toxicity: An Emerging Issue. Curr Med Chem (2018) 25(11):1327–39. doi: 10.2174/0929867324666170407125017 28403786

[125] Brieler J Breeden MA Tucker J. Cardiomyopathy: An Overview. Am Fam Phys (2017) 96(10):640–6. 29431384

[126] Figueroa MS Peters JI. Congestive Heart Failure: Diagnosis, Pathophysiology, Therapy, and Implications for Respiratory Care. Respir Care (2006) 51(4):403–12. 16563194

[127] Cooper LT Jr. Myocarditis. N Engl J Med (2009) 360(15):1526–38. doi: 10.1056/NEJMra0800028 PMC5814110 19357408

[128] Zhang J Zhang Q Chen X Zhang N. Management of Neoplastic Pericardial Disease. Herz (2020) 45(Suppl 1):46–51. doi: 10.1007/s00059-019-4833-4 31297544

